# Chromatin Unlimited: An Evolutionary View of Chromatin

**DOI:** 10.3390/epigenomes6010002

**Published:** 2022-01-02

**Authors:** Yasushi Hiraoka

**Affiliations:** Graduate School of Frontier Biosciences, Osaka University, Suita 565-0871, Japan; hiraoka@fbs.osaka-u.ac.jp

Chromatin is a fundamental and highly conserved structure that carries genetic and epigenetic information in eukaryotic cells [1]. When claiming evolutionary conservation, we often express it in terms of “yeasts to humans”; however, yeasts and humans belong to the same taxonomic supergroup, Opisthokonta, within a narrow range of eukaryotes [2]. This Special Issue, “Chromatin Unlimited”, aims to provide insights into the essential aspects of chromatin in a wider range of eukaryotes.

Chromatin is composed of DNA, histones, and other non-histone proteins. The minimum unit of canonical chromatin is the nucleosome, in which DNA is wrapped around the histone octamer [3]. Histones are among the most highly conserved proteins in eukaryotes [4]. However, several organisms have produced non-canonical forms of chromatin through evolution. A striking example is found in dinoflagellates, which express very low levels of histone proteins despite the fact that respective histone-coding genes are present in the genome and are transcribed [5,6,7,8]. In these organisms, histones are replaced by virus-derived non-histone proteins for the packing of DNA into chromatin. Another example of non-canonical chromatin stems from ciliated protozoans, which have two distinct nuclei (a somatic macronucleus and a germline micronucleus) that bear different types of chromatin, which are reorganized when the macronucleus is differentiated from the micronucleus [9,10,11]. It is possible that non-canonical forms of chromatin exist in more types of organisms than is presently known.

Non-canonical chromatins have also been found in more common organisms, such as sperm chromatin in mammals and erythrocyte chromatin in non-mammalian vertebrates. Except for limited regions, mammalian sperm chromatin contains no histones [12]. Sperm chromatin is packed with protamine, which is replaced by histones in an egg upon fertilization. Erythrocyte chromatin, which is transcriptionally inactive except for the globin gene locus, contains mainly histones as well as limited non-histone proteins [13]. Such examples have raised questions about the roles of histone proteins in chromatin functionality.

Additionally, it has been demonstrated through their in vitro reconstitution with purified proteins that chromosomes can be shaped with condensins and no histones [14,15]. Condensins are members of the structural maintenance of chromosomes (SMC) protein family. Because condensins are widespread in bacteria, archaea, and eukaryotes, chromatin with these SMC proteins may be of a more primitive form [16]. Therefore, although histones play a role in modulating the functions of chromatins, they may be dispensable in shaping the chromatin structures.

Finally, I wish to emphasize that chromatins can only be understood in light of evolution. Present-day organisms have survived selection through evolution. A phylogenic tree represents the evolutionary trails of present-day organisms originating from their ancestors. Evolution has never occurred directionally toward the branches of the tree and, instead, has been driven toward all unbiased directions and biased by the survival of the fittest. Thus, a phylogenic tree is a meshwork of the species left over after extinction removed the areas between the branches. We will never know the types of chromatin that existed in organisms that failed to survive. All we can do is comprehensively analyze and compare present-day organisms and trace them back to a common ancestor [17,18,19]. A deeper understanding of the non-canonical forms of chromatin will paradoxically shed light on the essential aspects of the most common canonical ones.
